# Development of a novel RANKL-based peptide, microglial healing peptide1-AcN (MHP1-AcN), for treatment of ischemic stroke

**DOI:** 10.1038/s41598-018-35898-z

**Published:** 2018-12-11

**Authors:** Munehisa Shimamura, Hironori Nakagami, Hideo Shimizu, Hideyuki Mukai, Ryosuke Watanabe, Takeshi Okuzono, Tomohiro Kawano, Yuka Ikeda, Hiroki Hayashi, Shota Yoshida, Nan Ju, Hideki Mochizuki, Ryuichi Morishita

**Affiliations:** 1 Department of Clinical Gene Therapy, Osaka University Graduate School of Medicine, Osaka, Japan; 2 Department of Health Development and Medicine, Osaka University Graduate School of Medicine, Osaka, Japan; 3 Department of Neurology, Osaka University Graduate School of Medicine, Centre of Medical Innovation and Translational Research (6th floor, Room 0612B), Osaka University, 2-2 Yamada-oka, Suita Osaka, 565-0871 Japan; 4 Department of Internal Medicine, Osaka Dental University, Osaka, Japan; 5 Tsukuba Laboratories, Nemoto Science Co., Ltd, Ibaraki, Japan; 6 Contract Research Department, Drug Development Solutions Center, Drug Development Solutions Division, Sekisui Medical Co., Ltd, Ibaraki, Japan

## Abstract

Although the regulation of post-ischemic inflammation is an important strategy to treat ischemic stroke, all clinical trials have failed to show its efficacy. To solve the problem, we previously developed a novel partial peptide of RANKL, microglial healing peptide 1 (MHP1), which could reduce ischemic injury by inhibiting Toll-like receptor (TLR) induced inflammation. However, optimization of the peptide was necessary to increase the stability and efficacies for clinical use. According to information gathered through HPLC/MS in serum, we have newly designed a series of modified MHP1 peptides and have found that N-terminal acetylation and C-terminal amidation in MHP1 (MHP1-AcN), can strengthen its anti-inflammatory effects and increase its stability with anti-osteoclastogenic effects. Anti-TLR activity was reported to be reduced in MHP1 when incubated at 37 °C for 24 hrs, but MHP1-AcN could keep the activity under the same condition. The therapeutic effect of MHP1-AcN was observed in transient ischemic stroke model at lower dose than MHP1. Importantly, MHP1-AcN did not affect thrombolytic effects of tissue plasminogen activator (tPA) and inhibited tPA-induced hemorrhagic transformation. These findings indicated that MHP1-AcN was stable and effective anti-TLR signal peptide and could be a promising agent for treating stroke patients receiving tPA and endovascular therapy.

## Introduction

Previously, we reported that the receptor activator of the nuclear factor-кB ligand (RANKL)/receptor activator of NFκB (RANK) was a novel signal for the reduction of TLR4-related inflammation in activated microglia and macrophages (M/M) in ischemic stroke^1^. As clinical trials targeting the classical inflammation pathways have failed to show the efficacy^2,3^, we speculated that stimulating this novel signal by recombinant RANKL (rRANKL) may be a promising new strategy for the treatment of ischemic stroke^1^. The stimulation of RANKL/RANK signal by systemic injection of recombinant RANKL, however, induced osteoclast differentiation and osteoporosis^4^ that are problematic in post-stroke patients.

To solve this problem, we developed a novel peptide “microglial healing peptide1 (MHP1)”, which is a partial peptide of RANKL, to inhibit anti-TLR4-induced inflammation without activating osteoclast^5^. The binding sites of RANKL for its receptor RANK responsible for osteoclastogenesis were not included in the MHP1 sequence, but other binding sites that are unrelated to osteoclastogenesis (DE and EF loops) were included. MHP1 did not affect osteoclast activation and also inhibited RANKL-induced osteoclast activation^5^. The sequence of MHP1 is “LMVYVVKTSIKIPSSHNLMKGGSTKNWSGN” and we demonstrated that “LMVYVVKTSIKIPSS” was a key region for anti-TLR-induced inflammation^5^. Importantly, MHP1 also inhibited TLR2, 7, 8-induced inflammations^5,6^, which are other important signals for DAMP in post-ischemic inflammation^7,8^.

In the transient middle cerebral artery occlusion (tMCAO) model, we showed that ischemic injury was reduced when MPH1 was injected either intracerebroventricularly 4 h, or, intravenously 4 or 6 h post-ischemia^5,6^. Fluorescein isothiocyanate-conjugated MHP1 revealed that MHP1 could penetrate the cerebral cortex in the ischemic hemisphere^6^, however, the stability of MHP1 and the degradation product in blood have not been clarified. Since unmodified synthetic peptide easily loses its activity due to rapid degradation within minutes by enzymes^9^, modification of MHP1 was required to increase the stability for clinical use.

Here we examined the stability and degradation product of MHP1 in mice serum using high-performance liquid chromatography-mass spectrometry (HPLC/MS). Based on the information gathered, we designed a series of modified MHP1 to improve effectiveness in their anti-inflammatory effects and the best peptide was screened using lipopolysaccharide Lipopolysaccharides (LPS)-stimulated MG6 cells. We further checked the pharmacokinetics and stability of selected peptides and examined its efficacy in the tMCAo model. The effects of the modified MHP1 on tissue plasminogen activator (tPA)-induced thrombolysis and cerebral hemorrhage were also examined because tPA is commonly used in the acute stage of ischemic stroke clinically.

## Results

### Designing of modified MHP1

**Figure 1:**
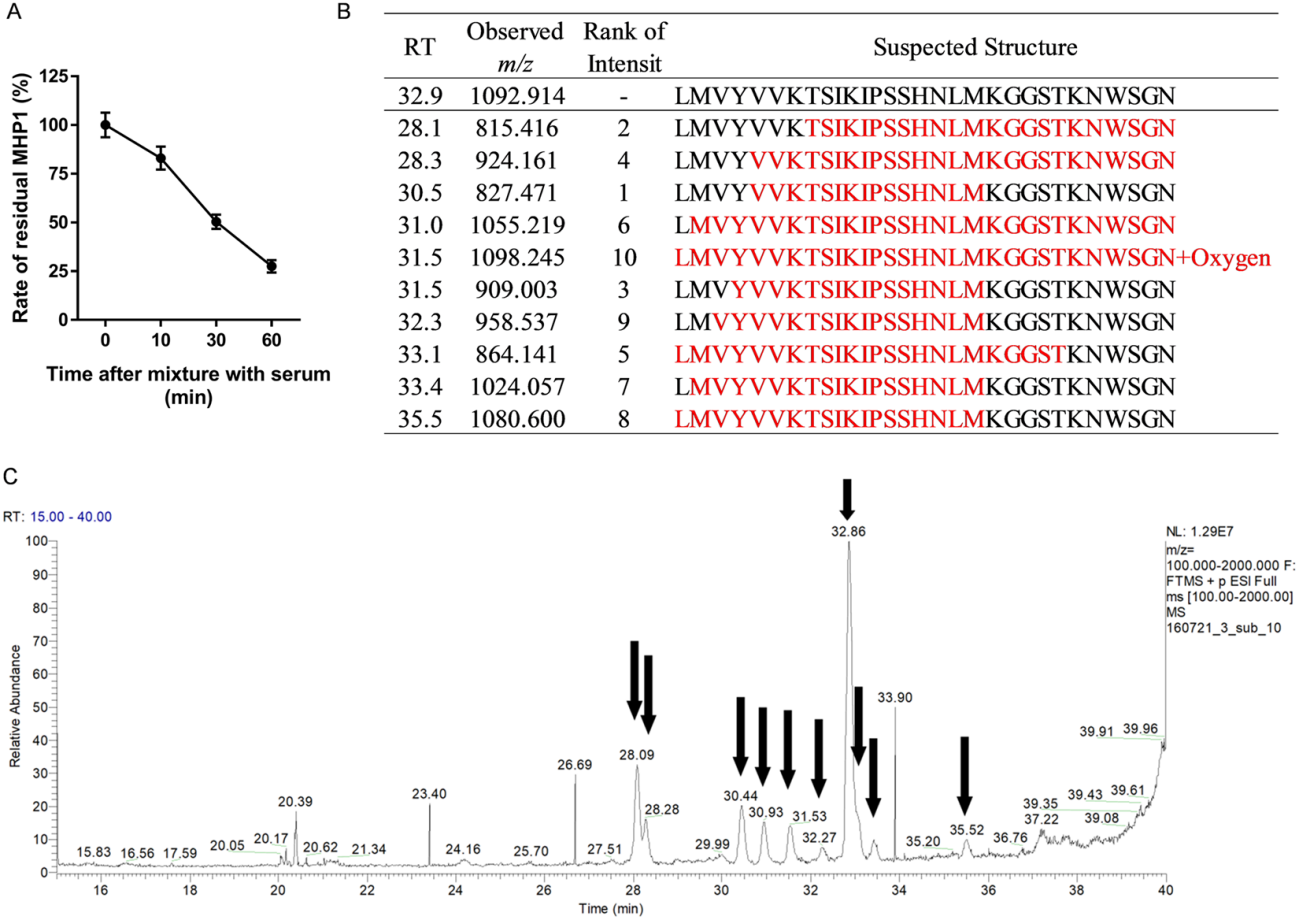
Stability and degradation product of MHP1 in mouse serum. (**A**) The residual MHP1 after addition to mouse serum was analyzed using HPLC. Although MHP1 was gradually degraded, 27.5 ± 3.2% remained after 60 min. (**B**,**C**) The degradation products were identified with mass spectrometry. The values of RT in (**B**) coincide with the arrowed time in (**C**). Colored sequences in (**B**) are degradation products.

First, we examined the stability of MHP1 in mouse serum using HPLC/MS with results of the analysis shown as a percentage of the residual MHP1. When MHP1 (2 mg/mL) was mixed with mouse serum to obtain a final concentration of 200 μg/mL MHP1, 83.0 ± 5.9%, 50.3 ± 3.6%, or 27.5 ± 3.2% remained intact at 10, 30, or 60 min, respectively (Fig. 1A). Although the peptide bonds of MHP1 were cleaved at the N-terminus (RT: 28.1, 28.3, 31.0), C-terminus (RT: 33.1, 33.5), and both termini (RT: 30.5, 31.5, 32.3, 33.4) (Fig. 1B,C), some degradation products, such as “LMVYVVKTSIKIPSSHNLMKGGSTKNWSGN-Oxygen”, “LMVYVVKTSIKIPSSHNLMKGGS”, or “LMVYVVKTSIKIPSSHNLM”, included the sequence “LMVYVVKTSIKIPSS”, which is a key region for anti-TLR-signaling^5^.

Based on the above results, we designed several peptides to avoid enzymatic degradation in blood. First, we substituted an amino acid with D-amino acid^10^. As the N-terminus “LMVYVVKTSIKIPSS” is the key region for the anti-TLR activity in MHP1 and degradation of this region completely loses its activity^5^, we designed 3 peptides with an amino acid in this region replaced by D-amino acid (MHP1-DN1, MHP1-DN2, MHP1-DN3 in Table 1, to avoid degradation in this region. In MHP1-DN1 and MHP1-DN2, Lys7 and Tyr4 were replaced by D-amino acid, respectively, as the amount of degradation product in this region ranked as the top 2 (28.1 or 30.5 mm). In MHP1-DN3, we substituted Met2 as methionine is generally easily oxidized^11^. We then designed MHP1-DC, where Met19 was replaced by D-amino acid. This was because MHP1 can be easily cut at Met19 (30.5, 31.5, 32.3, 33.4, 35.5 min) and deleting the C-terminus was reported to reduce its anti-inflammatory effects^5^. Finally, we designed MHP1 acetylated in the N-terminus (MHP1-Ac), amidated in the C-terminus (MHP1-N), or added both modifications (MHP1-AcN) to increase the biological stability^12^.

**Table 1:** Amino acid sequences of modified MHP1.

Name	Amino acid sequence
MHP1	LMVYVVKTSIKIPSSHNLMKGGSTKNWSGN
MHP1-DN1	LMVYVVkTSIKIPSSHNLMKGGSTKNWSGN
MHP1-DN2	LMVyVVKTSIKIPSSHNLMKGGSTKNWSGN
MHP1-DN3	LmVYVVKTSIKIPSSHNLMKGGSTKNWSGN
MHP1-DC	LMVYVVKTSIKIPSSHNLmKGGSTKNWSGN
MHP1-Ac	Ac-LMVYVVKTSIKIPSSHNLMKGGSTKNWSGN
MHP1-AcN	Ac-LMVYVVKTSIKIPSSHNLMKGGSTKNWSGN-NH_2_
MHP1-N	LMVYVVKTSIKIPSSHNLMKGGSTKNWSGN-NH_2_

Lower-case characters showed amino acids, which were replaced by D-amino acids.

### Effects of modified MHP1 in LPS-stimulated MG6 cells

**Figure 2:**
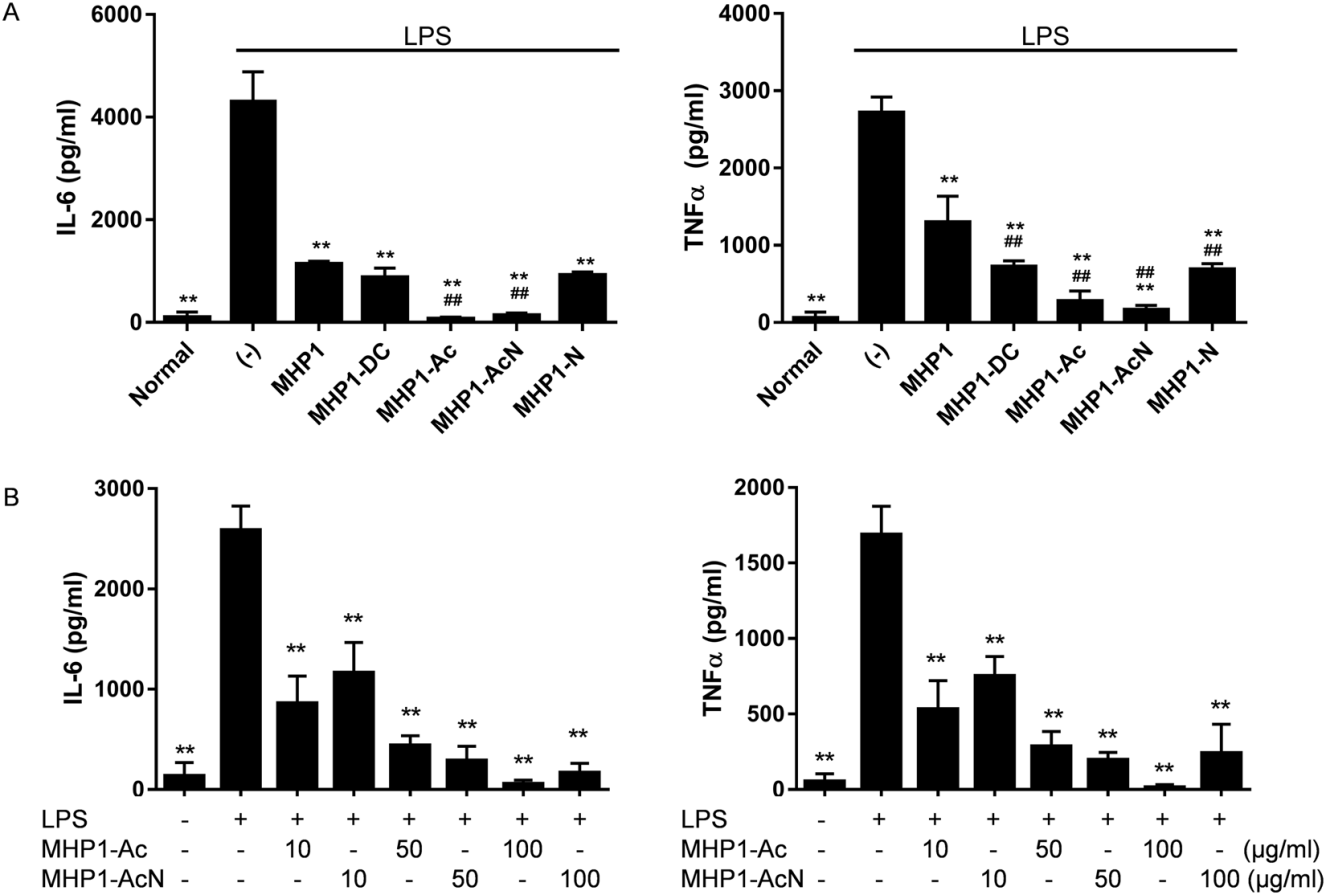
Effects of modified MHP1 in LPS-induced inflammation in MG6 cell. (**A**) Anti-inflammatory effects of modified MHP1 in LPS-stimulated MG6 cells. **P < 0.01 vs LPS-treated cells, ^##^P < 0.01 vs MHP1-treated cells. (**B**) Dose dependency of anti-inflammatory effects in MHP1-Ac and MHP1-AcN. **P < 0.01 vs LPS-treated cells.

**Figure 3:**
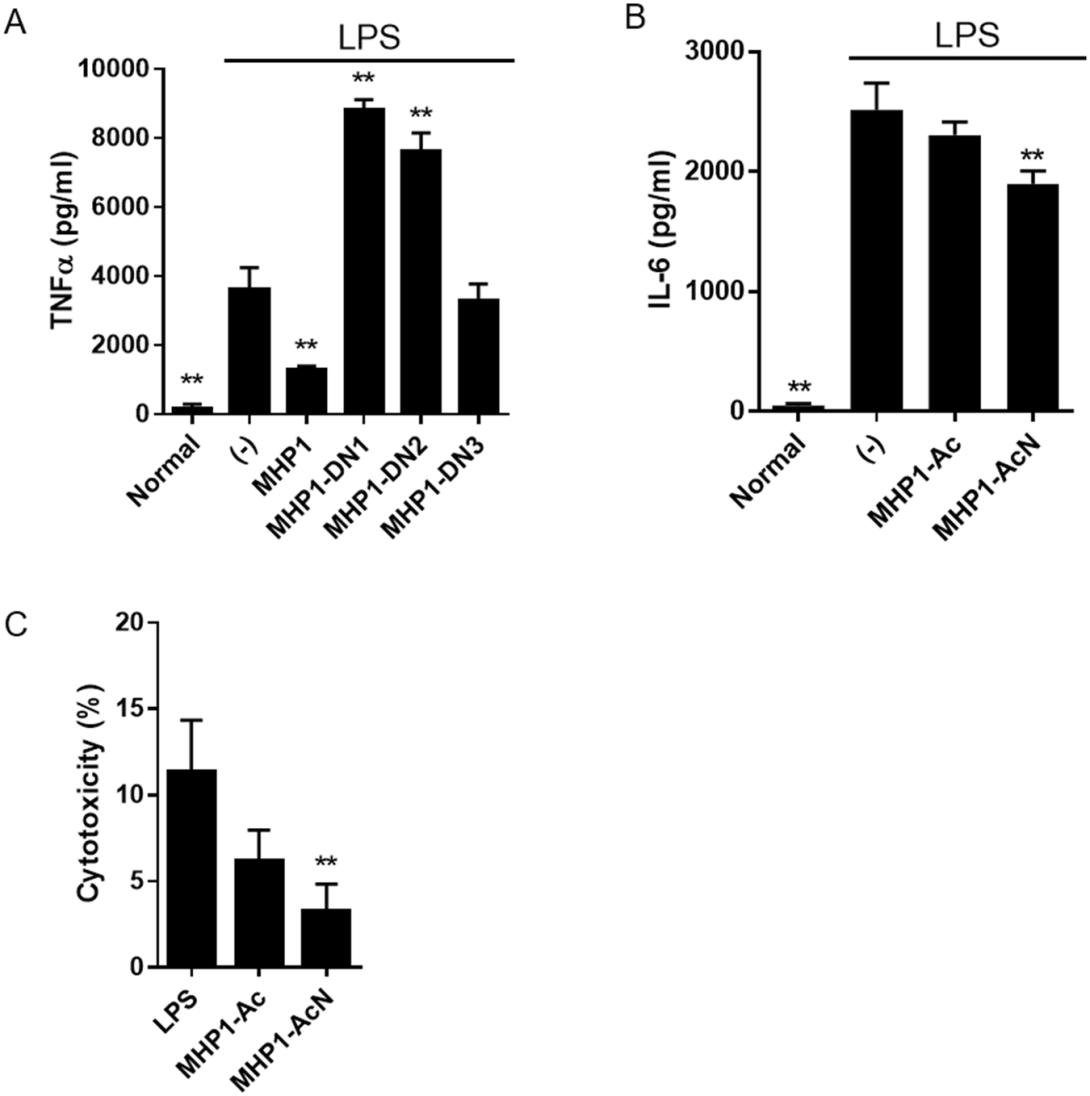
Effects of modified MHP1 in MG6 cells. (**A**) Effects of MHP1-DN1, MHP1-DN2, MHP1-DN3 on LPS-stimulated MG6 cells. Although MHP1-DN3 showed no influence on the expression of TNF-α, MHP1-DN1 and MHP1-DN2 augmented the expression of TNF-α. (**B**) Effects of 1 µg/ml of MHP1-Ac or MHP1-AcN in LPS-stimulated MG6 cells. (**C**) LDH assays in LPS-stimulated MG6 cells. **P < 0.01 vs LPS-stimulated cells without treatment.

We verified the effects of anti-TLR4-mediated inflammation in LPS-stimulated MG6 cells. MHP1-Ac and MHP1-AcN were more effective than MHP1 in the inhibition of IL-6 and TNF-α expressions (Fig. 2A). MHP1-DC and MHP1-N were also more effective than MHP1 in the reduction of the expression of TNF-α; this result was not replicated for IL-6 (Fig. 2A). MHP1-DN1, MHP1-DN2, and MHP1-DN3, however, lacked anti-inflammatory effects (Fig. 3A), and MHP1-DN1 and MHP1-DN2 augmented the expression of TNF-α. This indicated that the modification of the N-terminus of MHP1 results in a loss in its anti-inflammatory effects. As MHP1-Ac and MHP1-AcN proved to be the most effective peptides, they were the prime peptides of focus in the experiments to follow. Dose-dependent anti-inflammatory effects were observed both in MHP1-Ac and MHP1-AcN (Fig. 2B). Interestingly, anti-inflammatory effects were seen even in 1 µg/ml of MHP1-AcN; this was not seen in MHP1-Ac (Fig. 3B). To exclude the possibility that the anti-inflammatory effects were due to cell death, we examined an LDH assay (Fig. 3C). Cells treated with MHP1-Ac or MHP1-AcN did not increase cytotoxicity (Fig. 3C), indicating that the anti-inflammatory effects were not a result of increased cell death.

### Effects of MHP1-AcN in tMCAo model in mice

**Figure 4:**
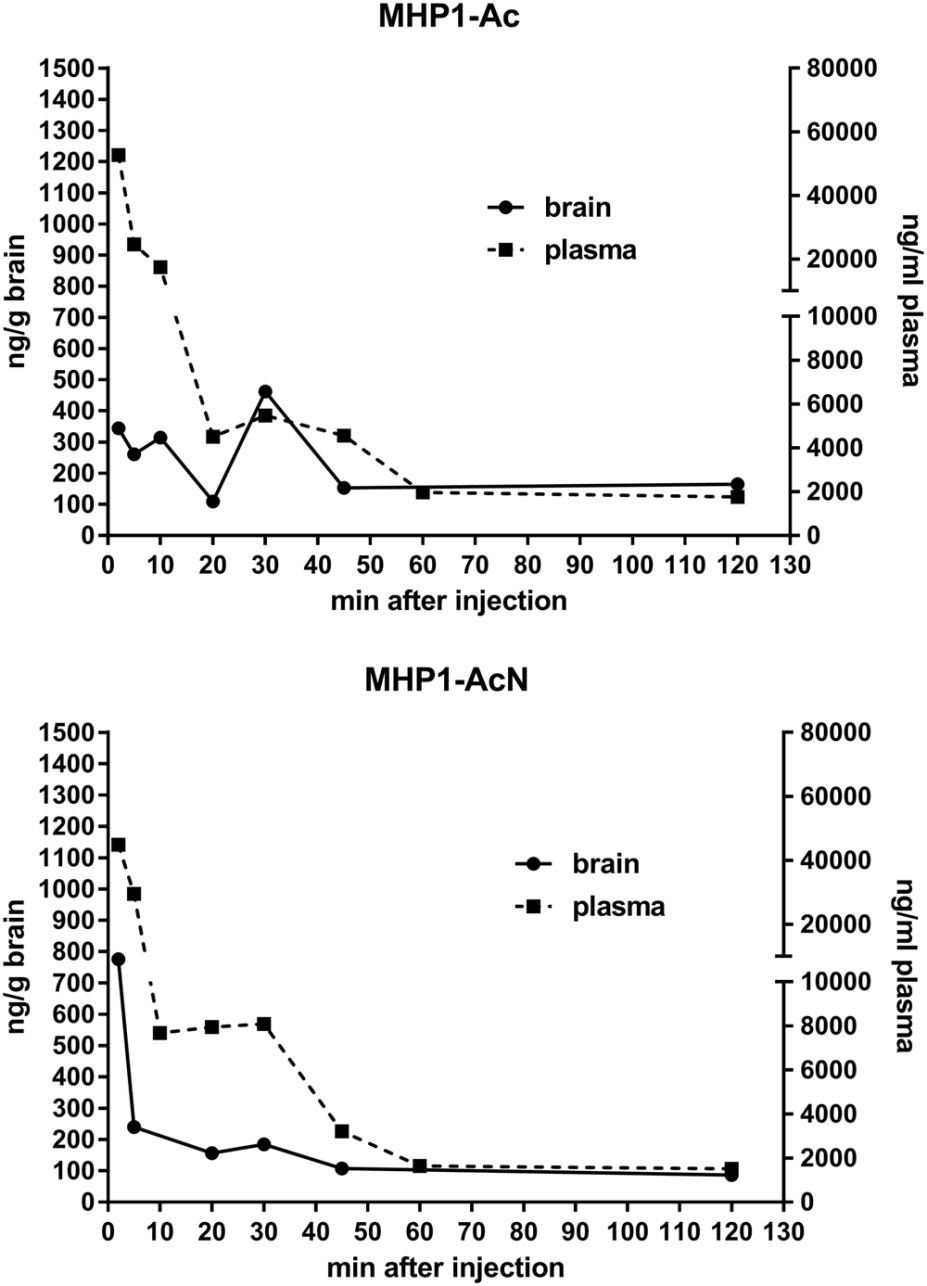
Pharmacokinetics of modified MHP1-Ac or MHP1-AcN after intravenous injection post-cerebral ischemia. MHP1-Ac or MHP1-AcN were intravenously injected 4 h after tMCAo. Although MHP1-Ac was quickly degraded in plasma, approximately 8000 ng/ml of MHP1-AcN was preserved at 30 min after injection.

To investigate their clinical applications, we evaluated the pharmacokinetics of MHP1-Ac and MHP1-AcN after intravenous injection beginning 4 h after tMCAo. LC/MS/MS analysis showed that around 8000 ng/ml of MHP1-AcN was intact 30 min post-injection while MHP1-Ac quickly degraded within 20 min (Fig. 4). In the brain, both MHP1-Ac and MHP1-AcN were detected until 2 h post-injection.

**Figure 5:**
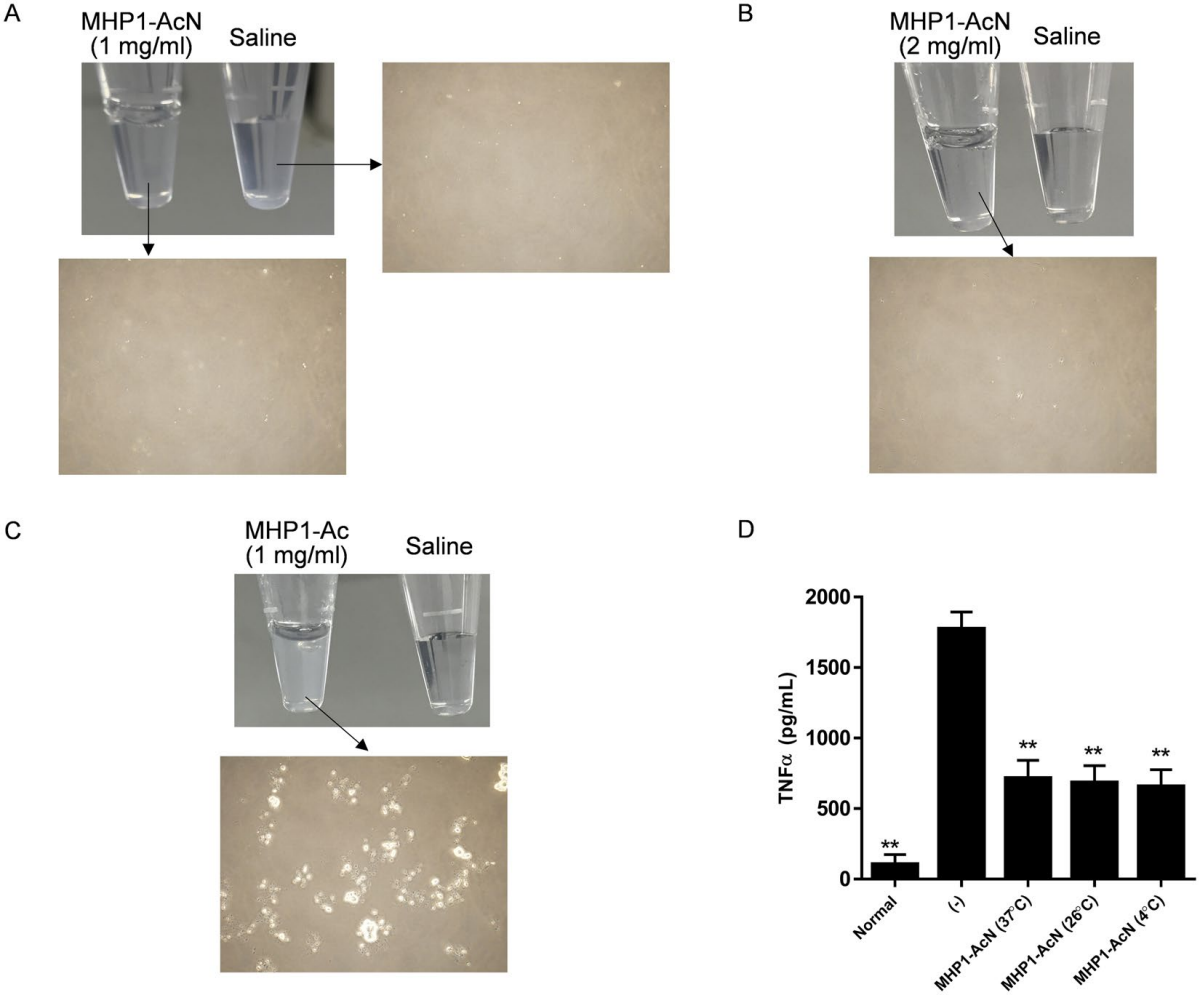
Stability of modified MHP1-Ac or MHP1-AcN in 0.45% saline incubated at 37 °C for 24 hours. MHP1-Ac and MHP1-AcN solution dissolved in 0.45% saline was incubated at 37 °C for 24 h. (**A**–**C**) typical images of solutions after 24 h. MHP1-AcN (1 mg/ml, **A**) and MHP1-AcN (2 mg/ml, **B**) solutions were clear, but MHP1-Ac solution showed sedimentation (**C**) Compared to MHP1-AcN incubated at 4 °C for 24 h, incubation in 37 °C for 24 h did not affect the anti-inflammatory effects (**D**). **P < 0.01 vs LPS-treated cells.

Since one problem of MHP1 for clinical use is the reduction of anti-TLR activity at body temperature, we checked the stability of MHP1-Ac and MHP1-AcN at 37 °C. MHP1-Ac and MHP1-AcN were easily dissolved in water, but MHP1-Ac experienced sedimentation when it was incubated in 0.45% saline at 37 °C for 24 h (Fig. 5C). On the other hand, sedimentation was not observed in MHP1-AcN at 1 and 2 mg/ml under the above conditions (Fig. 5A,B). When MHP1-AcN incubated in 4, 37, or 26 °C, which is a typical room temperature for continuous intravenous infusion with infusion solution bag, for 24 h was added to the medium containing MG6 cells stimulated with LPS, there were no significant differences in the expression of TNF-α (Fig. 5D), indicating that the anti-inflammatory effect of MHP1-AcN was not affected by temperature.

As MHP1 did not affect osteoclast differentiation, separately from RANKL^5^, we further checked the effects of MHP1-AcN on osteoclast differentiation (Supplementary Fig. S1). As expected, MHP1-AcN did not affect the osteoclast differentiation and inhibited RANKL-induced osteoclast differentiation.

Next, we evaluated the safety of MHP1-AcN in normal mice. Intravenous injection and continuous subcutaneous injection of MHP1-AcN with an Alzet pump showed no abnormalities in blood biochemistry at 24 h post-injection (Supplementary Fig. S2).

**Figure 6:**
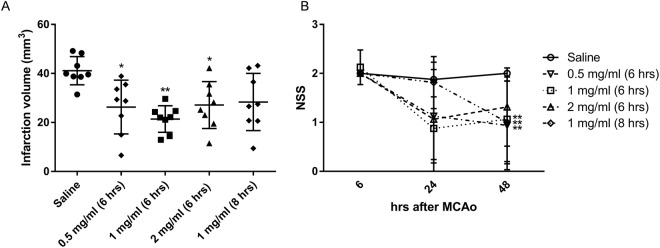
Therapeutic effects of MHP1-AcN after transient middle cerebral artery occlusion. (**A**) Infarction volume at 48 h after ischemia. MHP1-AcN began 6 or 8 h post-ischemia at various doses. (**B**) Temporal changes of neurological severity score after ischemic stroke. **P < 0.01, *P < 0.05 vs saline-treated mice.

We then examined whether MHP1-AcN would exhibit a therapeutic effect on ischemic injury in the tMCAo model. As we have previously shown that intravenous injection of MHP1 (150 μL, 2 mg/ml), started from 6 hrs after tMCAo and followed by subcutaneous injection with Alzet pump (200 μL, 2 mg/ml) for 24 h, is effective^6^, we studied whether a lower dose of MHP1-AcN was effective in reducing ischemic injury. As expected, MHP1-AcN was effective at 0.5, 1, and 2 mg/ml, with 1 mg/ml proving to be the most effective dose to reduce infarct volume (Fig. 6A). Neurological deficits were significantly improved in 0.5 and 1 mg/ml and there was a tendency of recovery in 2 mg/ml (Fig. 6B). As 1 mg/ml was the most effective dose in reducing infarct volume and neurological deficit, we further examined the cerebroprotective effects of MHP1-AcN (1 mg/ml) beginning 8 h after ischemia. There was a tendency of reduction in the infarct volume when compared to saline-treated mice (Fig. 6A). Neurological deficit was, however, significantly improved at 48 h after ischemia (Fig. 6B).

### Effects of MHP1-AcN on thrombolysis and hemorrhage with t-PA

**Figure 7:**
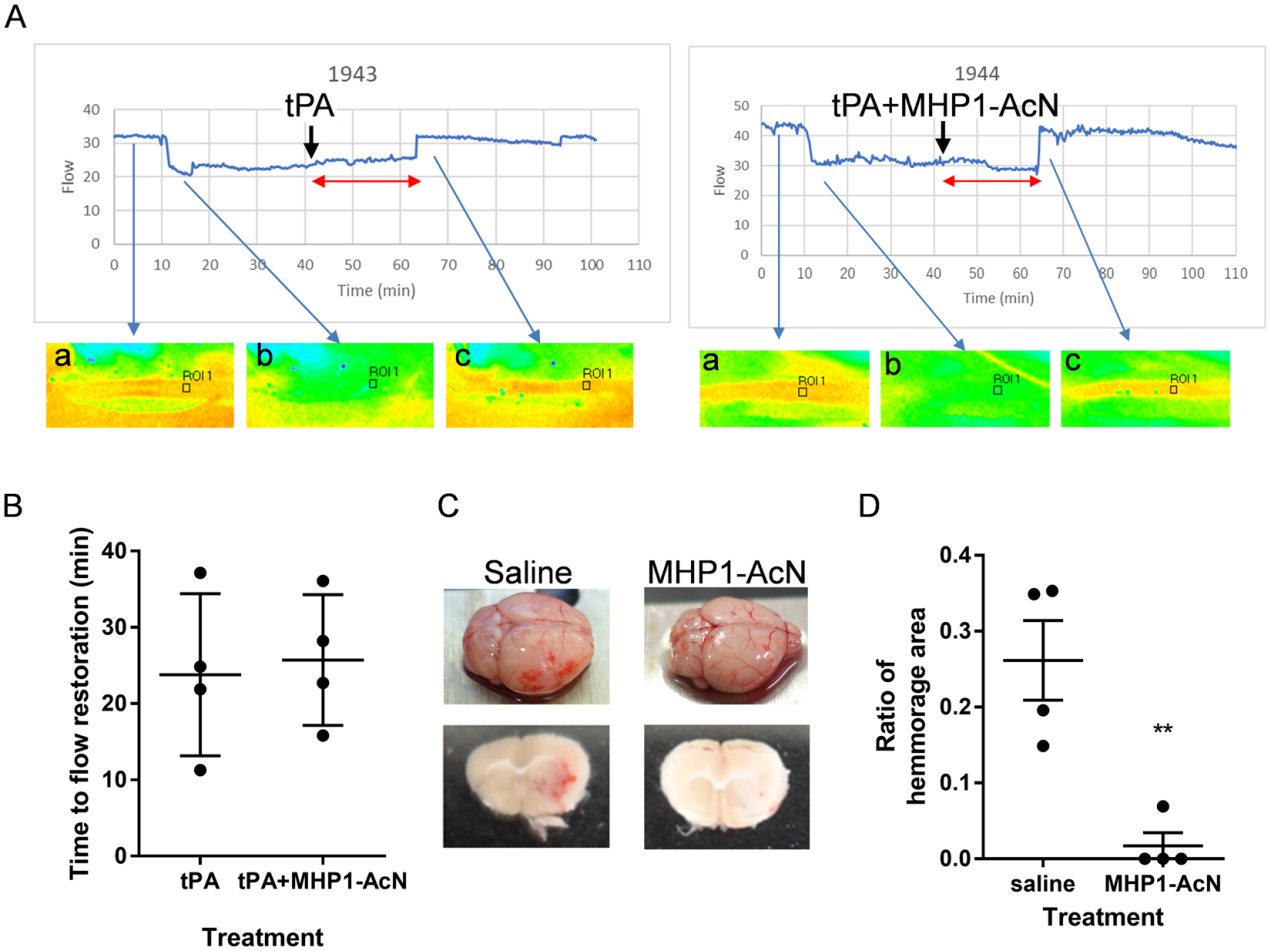
Influences of MHP1-AcN on the effects of tPA-induced thrombolysis and hemorrhage. (**A**) Typical flowmetry (upper) and typical laser speckle images (down) of FeCl_3_-induced thrombosis model in common carotid arteries (CCA). A filter paper soaked with FeCl_3_ was applied on the surface of CCA (a) followed by CCA occlusion (b). tPA or combination of tPA and MHP1 began 30 min after occlusion (black arrows) recovered blood flow (c). (**B**) The duration between starting of tPA and recanalization (red arrows in (**A**) were measured and quantified. (**C**) Typical images of tPA-induced hemorrhage. Co-treatment with MHP1-AcN inhibited hemorrhage. (**D**) Quantitative analysis of hemorrhagic region. MHP1-AcN reduced tPA-induced hemorrhage. **P < 0.01 vs saline.

Since tPA is the first-line of treatment in ischemic stroke, we examined the effects of MHP1-AcN on the action of tPA. First, we observed the effects of MHP1 on thrombolytic action of tPA using FeCl_3_-induced thrombosis model in CCA. This model has been previously demonstrated to be equivalent to thrombosis resolution using tPA in clinical studies^13^. When tPA was administered 30 min post-CCA occlusion, the clot was lysed approximately 20 min following tPA application (Fig. 7A,B). Administration of combined MHP1-AcN and tPA did not affect the time of recanalization (Fig. 7A,B), indicating that MHP1 did not affect the thrombolytic effect of tPA. We then examined whether MHP1-AcN affected tPA-induced hemorrhagic formation. Administering tPA immediately after reperfusion at 6 h in tMCAo model, hemorrhage transformation was observed 24 h after ischemia (Fig. 7C). Unexpectedly, mice treated with combination of t-PA and MHP1-AcN showed reduced formation of hemorrhage (Fig. 7C,D). These results indicated that MHP1-AcN could be administered with tPA.

## Discussion

Toward to the clinical trial, the present study demonstrated the successful design of a more effective and stable peptide, MHP1-AcN, to treat ischemic injury without affecting tPA-induced thrombolysis. Especially, MHP1-AcN was shown to effectively attenuate tPA-induced hemorrhage formation.

In general, most L-amino acid peptides with free N- and C-terminus are degraded in serum or plasma within minutes^12^. When compared to those peptides, unmodified MHP1 itself exhibits relative stability because 50% of MHP1 is preserved even 30 min after mixing with serum. Although MHP1 was degraded in the C-terminus and/or N-terminus in mice serum, some degradation products, such as “LMVYVVKTSIKIPSSHNLMKGGS” or “LMVYVVKTSIKIPSSHNLM” might still be effective owing to the inclusion of the “LMVYVVKTSIKIPSS”, which is key to anti-TLR-signaling^5^ as mentioned previously. The potential efficacy of the degradation products might explain the reason why the infarct volume was reduced at 48 h despite continuous injection for 24 h post-ischemic injury, in the previous study^6^. Other degradation products, such as “MVYVVKTSIKIPSSHNLM” which lacks leucine in the N-terminus, may not be effective as this amino acid in the N-terminus is necessary for anti-TLR-signaling activity^5^.

The experiment using MHP1-Ac and MHP1-AcN showed that the anti-inflammatory effects were enhanced by N-terminus acetylation. This might be a result of the key sequence of MHP1 being preserved owing to N-terminus acetylation, which generally makes the peptide resistant to protease degradation^14^. The difference in causing sedimentation at 37 °C for 24 h between MHP1-Ac and MHP1-AcN was dependent on the existence of C-terminal amidation, which has been reported to promote the stabilization of the secondary structure of the peptide^15^. We also showed that the activity of MHP1-AcN was not reduced after incubation at 37 °C for 24 h. This property is superior to MHP1, which loses its anti-inflammatory effects when incubated at 37 °C for 24 h^6^, and is important for clinical use. Although there are some concerns about safety in the modification, the acetylation and amidation in MHP1-AcN might be safe because some hormones and neuropeptides are naturally end-protected with acetylation and/or amidation to increase their stability^12^. The normal findings in blood test after the systemic injection support the speculation although further safety examination is required.

D-amino acid substitution in the C-terminus (MHP1-DC) also resulted in significant improvement of the anti-inflammatory effects. This may be due to the prevention of oxidization and degradation of methionine through its D-amino acid substitution. D-amino acid substitution in the N-terminus (MHP1-DN1, DN2), however, enhanced TLR4-induced inflammation. As the previous paper showed that aromatic D-amino acids acted as chemoattractant factors for human leukocytes through the G protein-coupled receptor^16^, D-amino acid substitution in this region may alter this property of MHP1 itself. This indicates that substituting the amino acid in this region should be avoided for further modifications.

The treatment with MHP1-AcN beginning 8 h post-ischemia showed variations in infarct volume compared to beginning at 6 h. As we have previously reported the expression of *RANK* mRNA was increased from 4 h after tMCAo and the peak of expression occurred at 12 h^1^, it was expected that delaying treatment until 12 h post-ischemia would have more effects than treatment started at an earlier time. This occurrence may be a result of neuronal cell death that has already begun 1.5 h after tMCAo and gradually increases until 24 h^17^. The inhibition of TLR-related inflammation at 8 h cannot prevent neuronal cell death in some case as the time would be too late.

Having no effect on tPA-induced thrombolysis by MHP1-AcN is important for clinical use. This is because the potential interactions between candidate treatments and thrombolysis in acute stroke trials have previously been highlighted^18^. In fact, a recent clinical study on subcutaneous IL-1 receptor antagonist injection failed to show its efficacy which may be due to the interactions with tPA^19^. Additionally, MHP1-AcN may expand the therapeutic time window of tPA as MHP1 is seen to inhibit tPA-induced hemorrhage. The mechanism of reducing tPA-induced hemorrhagic formation is rather unclear in this study, however, a recent study supports our findings^20^. In this study, TLR4^−/−^ mice had less hemorrhagic formation through reduced expression of MMP9 in vasculature, after delayed tissue plasminogen activator administration in thromboembolic stroke mice model^20^. Although expression of TLR4 was not determined in that report, other studies have shown that expression of TLR4 was increased in neurons, astrocytes, and microglia^21,22^ in intracerebral hemorrhage. In addition, TLR antagonist attenuated intracerebral hemorrhage-induced brain injury by reducing inflammation^22^. As microglial TLR4 plays the role of inducing endothelial activation^23^, MHP1-AcN, could suppress TLR4 signaling in microglia and inhibit tPA-induced hemorrhage formation through the inhibition of the endothelial activation.

In conclusion, we clarified the properties of modified MHP1-AcN and found that N-terminal acetylation and C-terminal amidation is suitable for enhancing the anti-inflammatory effects and stability of MHP1. Although further studies are necessary for the safety and efficacy in large animals such as monkeys^24^, MHP1-AcN may be a reliable therapeutic agent for reducing neurological deficits in ischemic stroke.

## Methods

### Peptide design and synthesis

Synthetic MHP1 (NH_2_-LMVYVVKTSIKIPSSHNLMKGGSTKNWSGN-COOH), MHP1-DN1 (NH_2_-LMVYVV-DLys-TSIKIPSSHNLMKGGSTKNWSGN-COOH), MHP1-DN2 (NH_2_-LMV-DTyr-VVKTSIKIPSSHNLMKGGSTKNWSGN-COOH), MHP1-DN3 (NH_2_-L-DMet-VYVVKTSIKIPSSHNLMKGGSTKNWSGN-COOH), MHP1-DC (NH_2_-LMVYVVKTSIKIPSSHNL-DMet-KGGSTKNWSGN-COOH), MHP1-Ac (Ac-LMVYVVKTSIKIPSSHNLMKGGSTKNWSGN-COOH), MHP1-AcN (Ac-LMVYVVKTSIKIPSSHNLMKGGSTKNWSGN-HN_2_), or MHP1-N (NH_2_-LMVYVVKTSIKIPSSHNLMKGGSTKNWSGN-NH_2_) was purchased from ILS, Inc (Ibaraki, Tsukuba, Japan), dissolved in 1 or 2 mg/ml ddH_2_O and stored at 4 °C until use (Table 1).

### HPLC/MS after mixing with mice serum

Mouse (CD-1(ICR)) serum was obtained from KAC (Kyoto, Japan) and stored below −15 °C until use. MHP1 (2 mg/mL in ultrapure water) was spiked in mouse serum on ice to obtain a final concentration of 200 μg/mL. The serum was incubated at 37 °C and aliquots of the reaction mixture collected at the incubation times 0, 10, 30, and 60 min. Each aliquot was deproteinized with the addition of an ice-cold solvent (acetonitrile: methanol: acetic acid = 500:500:1). The mixtures were left at −20 °C for 20 minutes and stored on ice. After the addition of 1% acetic acid, the mixture was vigorously vortexed and centrifuged (12000 × *g*, 4 °C, 10 min). The supernatant was injected into the HPLC system and analyzed by reversed-phase chromatography with a water–acetonitrile (0.1% trifluoroacetic acid) linear gradient elution. MHP1 was detected by ultraviolet light at 210 nm.

### Cell culture and enzyme-linked immunosorbent assay (ELISA)

MG6 cells were obtained from RIKEN BRC (Tsukuba, Japan)^25,26^. The MG6 cells were maintained in DMEM (Nakarai, Kyoto, Japan) supplemented with 10% FBS (Thermo Fisher Scientific, Waltham, MA, USA), 10 μg/ml insulin (Sigma-Aldrich, St. Louis, MO, USA) and 100 μM 2-mercaptoethanol (Sigma-Aldrich). These cells (1 × 10^5^ cells) were plated in 96-well plastic culture dishes. After overnight incubation, the medium was replaced with DMEM supplemented with 4% FBS. LPS (*Escherichia coli* 0111: B4; Sigma-Aldrich, St. Louis, MO, USA) and MHP1 or modified MHP1 was added to the medium, which was then harvested at 24 h after stimulation. In the experiment checking the stability at 26 or 37 °C, MHP1-Ac or MHP1-AcN was incubated at 26 or 37 °C for 24 h before adding to the medium.

The concentrations of TNF-α and IL-6 were measured using commercially available ELISA kits: TNF-α, Quantikine Mouse TNF-α ELISA Kit (R&D systems); IL-6, Quantikine Mouse IL-6 ELISA Kit (R&D systems).

Osteoclast differentiation was examined using a mouse osteoclast culture system obtained from Cosmo Bio Co. Ltd. (Tokyo, Japan). Mouse osteoclast precursor cells seeded in a 24-well plate were incubated with macrophage colony stimulating factor (50 ng/ml) and RANKL (50 ng/ml) with or without MHP1-AcN. After five days, osteoclast precursors stimulated with RANKL had fused with each other and became multinucleated TRAP-positive cells. Cells with more than ten nuclei were classified as large osteoclasts. The number of these cells in each group was counted with a phase contrast microscope (BZ-9000, Keyence, Tokyo) using BZII analyzer software (v1.42; Keyence, Tokyo).

### Surgical procedure

The plan of animal studies was approved by the Animal Committee of Graduate School of Medicine, Osaka University (25-029-012), and all animal experiments were carried out in accordance with the guidelines of Osaka University. All surgeries were performed using isoflurane and all efforts were made to minimize suffering. The C57Bl6/J mice were obtained from CLEA Japan, Inc. The transient middle cerebral artery occlusion procedure was described previously (1). Briefly, the mice were anesthetized with isoflurane (1.4%). The cerebral blood flow (CBF) was measured using a laser Doppler flowmeter (Unique Acquisition software; Unique Medical, Osaka, Japan). A 6.0 monofilament surgical suture was advanced into the internal carotid artery to obstruct the origin of the middle cerebral artery. The filament was left in place for 40 min before withdrawal. For all the mice, the rectal temperature was maintained at 37.0 ± 0.5 °C during surgery and recovery period (until the animals regained consciousness). Only animals that exhibited a typical reduction pattern and >82% reduction in the CBF during MCAo (in which CBF recovered by 30–80% after 5 min of reperfusion) and modified Bederson scale^27^ at 4 h after ischemia were included in the study. MHP1 (4 mg/ml in water) was diluted to 0.5, 1, or 2 mg/ml in 0.45% saline, and 150 μl of the MHP1 was injected intravenously at 6 or 8 h after the MCAo. MHP1 was subsequently injected subcutaneously for 24 hrs using Alzet mini-osmotic pump (2001D, DURECT Corporation, Cupertino, USA). The vehicle control for MHP1, 0.45% saline, was injected similarly.

The ischemic damage was evaluated at 48 h after MCAo in sections stained with cresyl violet. Coronal sections (12 μm thickness) were made at −1.4, −0.7, 0, 0.7 and 1.4 mm from the bregma, mounted on the stereomicroscope and photographed. The volume of the infarct was calculated by the sum of each area of all sections corrected by cerebral edema and multiplied by the thickness of the slices. In the experiment of tPA-induced hemorrhage, PE10-catheter was placed in the right internal jugular vein at 6 h after ischemia. The nylon suture was then removed and 10% of the MHP-AcN mixture (150 µg/mice) and tPA (10 mg/kg) were injected followed by continuous injection of residual mixture of MHP1-AcN and tPA using a syringe pump (UNIVENTOR 400 ANAESTHESIA UNIT, ZTN, MALTA). In the experiment of FeCl_3_ -induced thrombosis model in CCA, a piece of filter paper soaked in 7.5% FeCl_3_ was placed on the right CCA for 1 min and it was rinsed with saline. Blood flow was recorded by using a laser speckle blood flow imager (OMEGAZONE OZ-2, OMEGAWAVE, INC., Tokyo, Japan). tPA (10 mg/kg) or mixture of MHP1-AcN (150 μg/mice) and tPA was started to be administered at 30 min after CCA occlusion.

### Pharmacokinetics of MP1-Ac and MHP1-AcN after i.v. injection in mice

MHP1-Ac or MHP1-AcN (2 mg/ml, 10 ml/kg in 0.45% saline) was administered intravenously 4 hrs after tMCAo and mouse plasma was collected at 5 min, 10 min, 20 min, 30 min, 45 min, 1 h, and 2 h after injection. Mouse plasma (C57BL6J, 10 µL) and water/phosphoric acid (24:1, v/v) (Solution B, 500 µL) were mixed together to prepare the plasma samples. The samples (510 µL) were then analyzed using LC/MS/MS to determine MHP1-Ac or MHP1-AcN in plasma. Mouse brain was weighed and mixed with a 4-fold volume of solution B and the mixture was homogenized to prepare the brain samples. The specific gravity of solution B was assumed to be 1. The brain samples (10 µL) were analyzed using LC/MS/MS to determine MHP1-Ac or MHP1-AcN in brain. MHP1-Ac and MHP1-AcN, and MHP1-N (internal standard), in the plasma samples and brain samples, were extracted using Oasis HLB μElution Plate (Waters). Chromatographic analysis was performed on an InertSustain C18 (2.1 mm I.D. ×50 mm, 3 µm, GL Sciences) column using the mobile phase A, water/formic acid (1000:1, v/v), and the mobile phase B, acetonitrile/formic acid (1000:1, v/v).

### Statistical analysis

All values are expressed as the mean ± standard deviation (SD). Multiple comparisons were evaluated by analysis of variance (ANOVA) followed by Dunnett’s multiple comparison test. Two groups were compared using the unpaired t-test. Differences were considered significant when *P* < 0.05. Two-way ANOVA followed by Dunnett’s multiple comparison test was performed in neurological severity score. Differences were considered to be significant at *P* < 0.05. All statics were calculated using GraphPad Prism software version 6.07 (GraphPad, Inc., San Diego, CA, USA).

## Electronic supplementary material

Supplementary Information

## Data Availability

The data that support the findings of this study are available from the corresponding author upon reasonable request.
