# Supplementary material for: Development of a novel RANKL-based peptide, microglial healing peptide1-AcN (MHP1-AcN), for treatment of ischemic stroke
Source: Sci Rep. 2018 Dec 11;8:17770. doi: 10.1038/s41598-018-35898-z (PMC6290023; doi:10.1038/s41598-018-35898-z)
Supplement: Supplementary file 1 — Supplementary Information [file 41598_2018_35898_MOESM1_ESM.pdf]

Development of a novel RANKL-based peptide, microglial healing peptide1-AcN (MHP1-AcN), for treatment of ischemic stroke

Munehisa Shimamura[1,2*], Hironori Nakagami[2], Hideo Shimizu[3], Hideyuki Mukai[5], Ryosuke Watanabe[5], Takeshi Okuzono[6], Tomohiro Kawano[1,2], Yuka Ikeda[1], Hiroki Hayashi[1], Shota Yoshida[1], Nan Ju[1], Hideki Mochizuki[2], Ryuichi Morishita[4*]

1. Department of Health Development and Medicine, Osaka University Graduate School of Medicine, Japan
2. Department of Neurology, Osaka University Graduate School of Medicine, Japan
3. Department of Internal Medicine, Osaka Dental University, Japan
4. Department of Clinical Gene Therapy, Osaka University Graduate School of Medicine, Japan
5. Tsukuba Laboratories, Nemoto Science Co., Ltd, Japan
6. Contract Research Department, Drug Development Solutions Center, Drug Development Solutions Division, Sekisui Medical Co., Ltd, Japan

*Corresponding authors: Ryuichi Morishita[1], Munehisa Shimamura[2,3]

[1]Department of Clinical Gene Therapy, [2]Department of Health Development and Medicine and

[3]Department of Neurology, Osaka University Graduate School of Medicine, Centre of Medical Innovation and Translational Research (6th floor, Room 0612B), Osaka University, 2-2 Yamada-oka, Suita, Osaka 565-0871, Japan

Tel: +81-6-6210-8359; Fax: +81-6-6210-8360

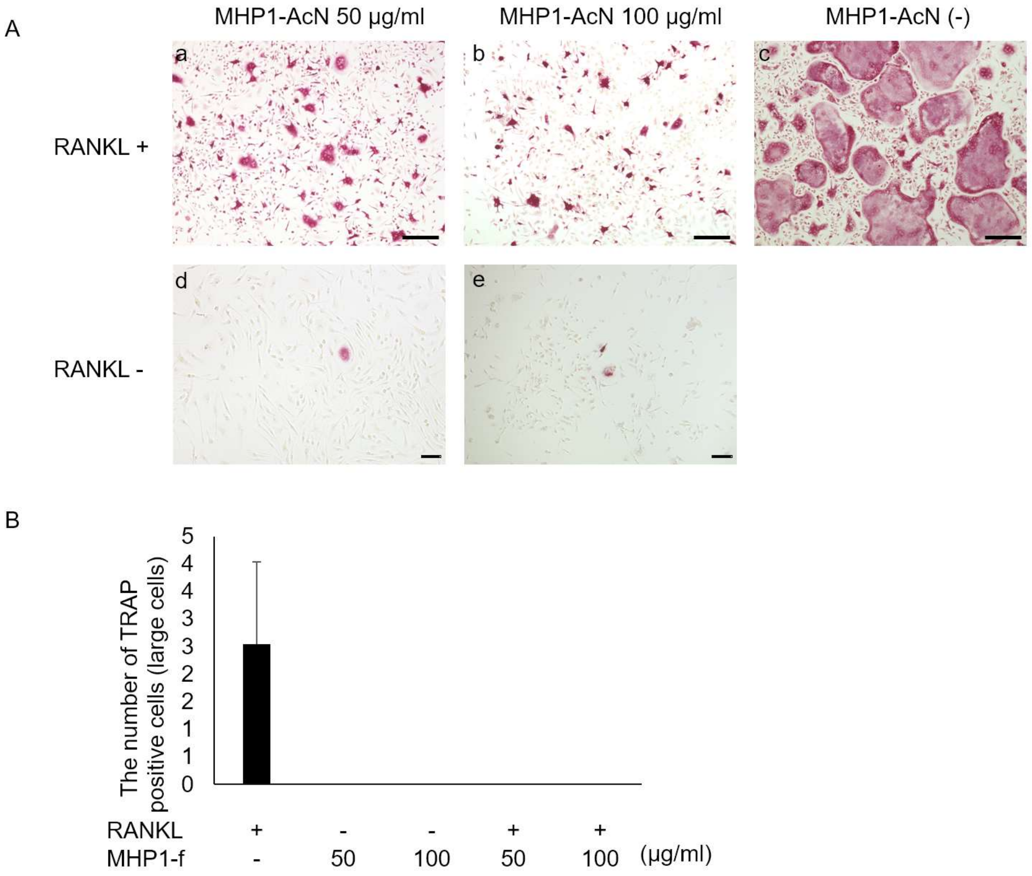

Supplementary Figure S1. Effects of MHP1-AcN on primary osteoclast precursor cells

Typical images of TRAP staining (A) and quantitative analysis of TRAP positive large cells (B). The osteoclast precursor cells were treated with combination of RANKL and MHP1-AcN (a, b), RANKL without MHP1-AcN (c), or MHP1-AcN without RANKL (d, e). Osteoclast precursor cells were differentiated into osteoclast after RANKL treatment (c), but MHP1-AcN did not induce the differentiation (d, e). Also, MHP1-AcN inhibited RANKL-induced osteoclast differentiation (a, b).

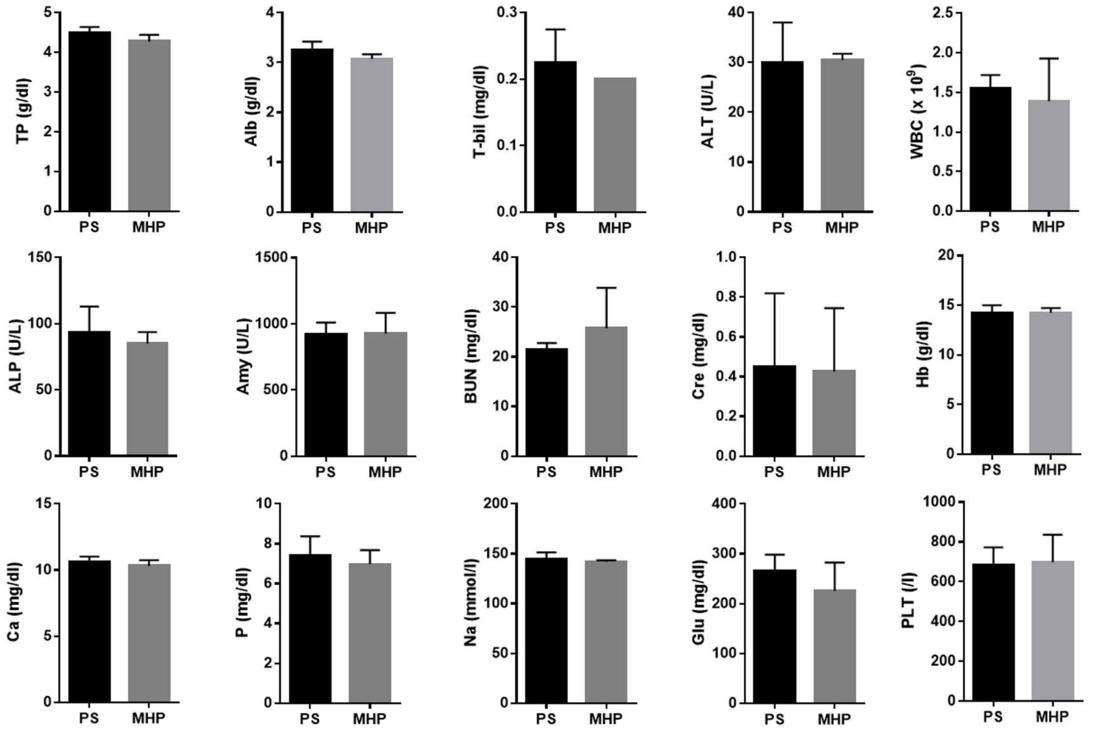

Supplementary Figure S2. Blood biochemistry after intravenous and continuous subcutaneous injection of MHP1-AcN in normal mice

MHP1 (1 mg/ml) was injected intravenously followed by continuous subcutaneous injection for 24 hrs. Significant differences were not seen between saline- and MHP1-AcN-treated mice.
